# Current News

**Published:** 1891-04

**Authors:** 


### Current News.

### TRACHEOTOMY SAFE AND QUICK.

H. H. SCHUHMANN, D.D.S.

DR. L. OLIVIERI, of Genoa, Italy, has invented an ingenious and novel instrument for the rapid and safe performance of tracheotomy. The main instrument consists of a sharp hook, grooved on its back to the extreme point and carrying on its rear-end a very small arm or lever, which latter carries a very light delicate windwheel. The instrument is intended to be used as follows: The point (of the sharp hook) is inserted into the trachea just below the cricoid cartilage, and the moment it pierces the trachea the air rushes along the groove and sets in rapid motion the little windwheel, thus informing the operator the instant the instrument reaches the proper place. A deceptive or false incision is thus rendered impossible at a time when the saving of a few minutes or even seconds are of the highest value in the saving of a human life. The more exhausted the patient, the feebler is the current of expired air, and hence the value of the delicate windwheel in these cases. By introducing a probe-pointed bistoury into the groove on the back of the hook and slipping it down into the wound, using the groove as a director, the opening becomes sufficiently enlarged for the next step in the operation. The hook and knife are now withdrawn, and the other instrument belonging to Dr. Olivieri's set is introduced into its place; it is a combined dilator and conductor, and is used to introduce the canula. This conductor is so constructed that it can be readily passed into a very narrow slit, which it dilates by giving it a half-turn. It places the canula in its proper place and leaves it there. The conductor is so ingeniously built that, in case there should be the slightest delay during this part of the operation, the patient can breathe freely through it until it is extricated and the canula remains in its place. Both conductor and canula are made of three different sizes, suitable to patients of different ages. When we remember that tracheotomy is an operation that has frequently to be performed in great haste to save a patient from imminent death, it will be seen that an instrument that makes it possible to perform it with certainty with three rapid motions is worthy of the attention of all oral surgeons and physicians. It is a difficult matter to explain mechanical appliances without illustrations, but if these lines are not fully understood, Dr. Olivieri will gladly give any information desired, at his office, located at 242 Wabash Avenue, Chicago.

---

On the line of the New York and New Haven Railroad, just outside the former city, a huge advertising sign-board reads as follows:

DR. ——— ———
HAS EXTRACTED OVER 500,000 TEETH,
ABSOLUTELY WITHOUT PAIN,
DENTISTRY { in all its branches. } FINE WORK ONLY.
57 WEST 42D ST., NEW YORK CITY.
9 A.M. to 5 P.M.—7 P.M. to 8 P.M.

Over five hundred thousand teeth torn from the jaws of his fellow-beings by one dentist alone! and, in addition to this wholesale extraction of teeth, the industrious and indefatigable doctor attends to "dentistry in all its branches" (presumably *roots* as well), and promises "fine work only" in addition.

Half a million teeth extracted "absolutely without pain" implies that every one of them were removed for patients while under the influence of some anæsthetic agent. Although it seems incredible that a single individual could administer anæsthetics and extract such an immense number of teeth in the practice of a lifetime, yet here we are assured in glaring capitals that "Dr. ——" has already exceeded that number, besides attending to "all branches of dentistry," and doing "fine work only." No wonder that the doctor is obliged to operate by gas-light as well as by daylight. It is a wonder if, under the pressure of these varied and multitudinous duties, the doctor gets time to give even a glance at any of the condemned teeth, in order to ascertain if it be not possible to treat and save them for a long period of usefulness.

But why advertise the number of teeth irretrievably lost? Does it signify a lack of ability to preserve these useful members of the human organism when attacked by caries; or are many of them removed with a view of replacing them with artificial dentures, and so keep active the mechanical branch of "fine work only?" Perhaps the "mighty dollar" is so large as to cloud the vision even if it does not veil the conscience. A half million teeth extracted and anæsthetics administered in each case would, at average charges, yield over half a million dollars. Then the profits from the "fine work" of "all other branches" in the line of dentistry (if also done by wholesale) would naturally be several times greater, making an aggregate perhaps of over two million dollars! What a temptation for a rising young man to become a dentist!

What can be the moral effect on public opinion of such roadside advertisements? The dentist, like the oculist, general surgeon, or family physician, is supposed to be a conservator of the human body; not to dismember it or remove any portion possible to save. If surgery means simply to amputate legs, arms, etc., it is pretty harsh surgery, to say the least; and if dentistry means simply to extract teeth by wholesale, and manufacture artificial substitutes, then its benefits to humanity are likewise very limited. C. E. F.

---

ILLINOIS STATE DENTAL SOCIETY.—The Twenty-seventh Annual Meeting will be held at Bloomington, beginning Tuesday, May 12, 1891, and continuing four days.

GARRETT NEWKIRK,
*Secretary.*

### CENTRAL DENTAL ASSOCIATION OF NORTHERN NEW JERSEY.

Newark, N. J., February 21, 1891.

The following officers and members of the Executive Committee were elected for the ensuing year, February 16, 1891.

President, C. W. F. Holbrook, D.D.S., Newark; Vice-President, H. Iredell, D.D.S., New Brunswick; Secretary, S. S. Hawley, D.D.S., corner of Warren and Thirteenth Streets, Newark; Treasurer, Charles A. Meeker, D.D.S., 29 Fulton Street, Newark.

*Executive Committee.*—George E. Adams, D.D.S., Chairman, South Orange; W. L. Fish, D.D.S., Newark; R. M. Sanger, D.D.S., East Orange; B. F. Luckey, D.D.S., Paterson; S. C. G. Watkins, D.D.S., Montclair.

S. S. Hawley, D.D.S.,
*Secretary.*

---

Dental Society of the State of New York.—The next annual meeting of the New York State Dental Society will be held at Albany, N. Y., May 13 and 14, 1891.

This will be the most interesting meeting held in years, as matters of vital importance to the dental profession will be presented. Reserve the above dates and attend. Head-quarters, Delavan House.

W. W. Walker, *President.*
F. T. Van Woert, *Secretary.*

---

Chicago, March 12, 1891.

The Executive Committee have decided on Saratoga Springs as the next place of meeting of the American Dental Association, commencing first Tuesday in August, 1891.

It is hoped by the committee that each society will send delegates, that we may have a full representation from all parts of the country.

Programme and arrangements to be announced later.

J. N. Crouse,
*Chairman Executive Committee.*

AMERICAN MEDICAL ASSOCIATION, SECTION OF ORAL AND DENTAL SURGERY.—The Forty-second Session of the American Medical Association will be held in Washington, D. C., on Tuesday, Wednesday, Thursday, and Friday, May 5, 6, 7, and 8, 1891, commencing on Tuesday, at 11 o'clock A.M.

The following is a list of essayists (with subjects), who have promised to prepare papers for the Section of Oral and Dental Surgery:

1.—Address of the Chairman of Section......... Dr. Eugene S. Talbot.
2.—Adenoid Growth.................................. Dr. W. H. Atkinson.
3.—Treatment of Fractures of the Maxilla.... Dr. Wm. Carr.
4.—Genesis of Contour Fillings. Illustrated... Dr. Geo. S. Allan.
5.—The Teeth of Invertebrate Animals......... Dr. A. H. Thompson.
6.—A Study in Comparative Dental Anatomy.. Dr. Wm. H. Potter.
7.—Rheumatic and Gouty Diathesis as manifested in Diseases of the Peridental Membrane.................................... Dr. John S. Marshall.
8.—Dental Infirmary Patients; the Use and Abuse of Dental Charity..................... Dr. Richard Grady.
9.—Growth of the Cementum...................... Dr. R. R. Andrews.
10.—Remarks on Incipient Necrosis and Caries.. Dr. J. Williams.
11.—Choice of Therapeutic Filling Materials... Dr. W. A. Alport.
12.—.................................................... Dr. J. Taft.
13.—Thorough Dentistry *vs.* Partial Dental Surgery.......................................... Dr. J. Y. Crawford.
14.—.................................................... Dr. Thos. Fillebrown.

Other members who desire to read papers before this Section, should, as required by the by-laws, forward the paper, or its *title* and *length*, to the Chairman, Dr. Eugene S. Talbot, 125 State Street, Chicago, Ill., one month before the meeting.

HENRY W. MORGAN,
*Secretary.*

NASHVILLE, February 23, 1891.

---

THE Second Annual Meeting of the Dental Protective Association of the United States was held at the Grand Pacific Hotel, Chicago, December 16, 1890. The president, Dr. Crouse, called the meeting to order, and spoke as follows:

Our second year closes full of encouragement. Last year at our First Annual Meeting we had represented, by proxy and in person, six hundred and forty-eight members. This our Second Annual Meeting is represented by nearly fifteen hundred members, showing that the membership has more than doubled during the year.

We have driven the Tooth Crown Company from Milwaukee, where they had commenced suits against five members. Our attorneys entered a motion asking the court to set and limit the time when all the testimony should be presented by the Crown Company. After hearing the arguments of the counsel on both sides, the court limited the time to twenty days, and before it was up the Tooth Crown Company withdrew all the suits at their own cost. A similar move in Baltimore, where six of our members had been sued, and we had taken charge of their suits, caused the Crown Company to withdraw all these suits at their own cost. This demonstrates the correctness of what we have all along claimed,—that the International Tooth Crown Company did not dare to enter into a fair contest as to the validity of their patents.

They have now commenced suits in New York, and answers will be filed in due time. We accept licensees on the same terms as others, and afford them the same protection, with the exception of a few licensees located in a very limited portion of the country.

The Dental Protective Association has been giving absolute protection to the entire dental profession, while, thus far, less than one-tenth of the whole number has joined our Association.

We want every member of the profession to unite with us. Ten dollars is but a trifle for each dentist to pay for the protection and benefits he receives. Think of fifteen thousand dentists in one association! Who can estimate the great good that such an organization can accomplish. Is it a dream? No! I expect to live to see it. It only depends on the amount of exertion the present fifteen hundred members put forth. Let every member during the year get three new members. It will be an easy way to treble our membership, which is the great work of the coming year.

We have saved the dental profession a million of dollars the past year and, better still, saved many of its members the humiliation of signing a license that robs a man of his manhood.

I want a committee appointed from this Association to examine our books and methods of doing the work.

On motion, Drs. Gilmer, Fernandez, and Ames were selected.

The election of a Board of Directors resulted in J. N. Crouse, T. W. Brophy, and E. D. Swain being elected their own successors. After further routine business the meeting adjourned.

J. N. Crouse, *President.*
E. D. Swain, *Secretary.*

### REPORT OF THE COMMITTEE ON DENTAL PROTECTIVE ASSOCIATION.

After a careful examination of the management and accounts of the Dental Protective Association of the United States for the year to date, we have come to the conclusion that it is being conducted carefully, economically, and with good judgment.

We know positively that the chairman is giving to this work much thought and time, which is the same as money to him; that during the year he has attended several dental meetings in the interest of the Association; that he has devoted much time and labor to organizing the profession and in attending to its litigation, and that he has done all this entirely at his own expense and without one dollar of cost to the Association.

The entire office expense of the Association for the year amounts to two hundred and forty dollars ($240.00), and that for clerical help.

The Dental Protective Association is saving hundreds of thousands of dollars annually to the profession, and we believe it to be the duty of every practising dentist to assist its grand work by paying the membership fee and becoming identified with it.

It is certainly unjust that a few should bear the entire expense for the benefit of the whole profession, and it is demoralizing to those who quietly accept the fruit of this injustice.

We would further recommend that, as soon as the management think it advisable, after due notice to the profession, the books be closed, and the protection of the Association be withheld from non-members.

Thos. L. Gilmer,<br>

E. M. S. Fernandez, } *Committee.*<br>

W. B. Ames,

---

### RESOLUTIONS ADOPTED BY THE SAN FRANCISCO DENTAL ASSOCIATION, OCTOBER, 1890.

Whereas, Dr. J. N. Crouse, of Chicago, Ill., the chairman of the Dental Protective Association of the United States, is personally known by the president and other members of the San Francisco

Dental Association to be an honest, earnest, and enthusiastic worker for the good of the profession; therefore be it

*Resolved,* That this Association endorse the methods of Dr. Crouse in conducting the Dental Protective Association, and strongly urge every dentist of the Pacific Coast to become a member of said Association; and be it also

*Resolved,* That a copy of this resolution, signed by the president and secretary, be forwarded to Dr. Crouse, with permission to insert it in each circular that he sends to this coast.

THOS. N. IGLEHART, *President.*
CHAS. E. POST, D.D.S., *Recording Secretary.*

---

### COLLEGE COMMENCEMENTS.

THE Philadelphia Dental College held its Twenty-eighth Annual Commencement, February 26, 1891, at the Academy of Music, in the City of Philadelphia.

The matriculates numbered 315, and the graduates 146. No absentees.

The address to the graduates was given by Professor J. Foster Flagg, and the valedictory by Charles L. Ziegler, D.D.S.

On the following evening, February 27, the Pennsylvania College of Dental Surgery held its commencement at the same place.

The matriculates numbered 251, with 18 absent; the graduating class, 94.

Professor C. N. Peirce delivered the valedictory.

The audiences were large on both occasions.

The Association of Faculties requires that absentees during the session shall be marked on the published list with an asterisk (*). The absence of this in the Philadelphia Dental College list indicates either a remarkable attendance on the part of the matriculates or a failure to comply with the rule.

Kansas City Dental College Commencement, March 10, 1891. Graduates, 43; matriculates, 107.

Ohio College of Dental Surgery Commencement, March 11, 1891. Graduates, 75; matriculates, 210.

Missouri Dental College Commencement, March 12, 1891. Graduates, 28; matriculates, 90.

The Eighth Annual Meeting of the Maryland State Dental Association, just closed at Baltimore, elected the following officers for 1891:

Cyrus M. Gingrick, D.D.S., President; B. Holly Smith, M.D., D.D.S., First Vice-President; Bernard Myer, D.D.S., Second Vice-President; F. F. Drew, D.D.S., Corresponding Secretary; W. W. Dunbracco, D.D.S., Recording Secretary; Thos. H. Davy, D.D.S., Treasurer.

*Executive Committee.*—C. C. Harris, D.D.S., Jno. C. Uhler, M.D., D.D.S., G. Marshall Smith, D.D.S.

A resolution was adopted to include the name of Horace H. Hayden in the memorial about to be established to the memory of Chapin A. Harris.

W. W. Dunbracco,
*Secretary.*

Baltimore, February 12, 1891.

---

### HARVARD ODONTOLOGICAL SOCIETY.

The Thirteenth Annual Meeting and dinner of the Harvard Odontological Society was held at Young's Hotel, Boston, on Saturday, February 28, twenty-four members in attendance. The meeting was called to order at 5.30 p.m., by the President, Dr. William P. Cooke, of Boston; and after listening to the annual reports and the transaction of regular business, the Society gave its attention to the orator of the evening, Dr. Henry L. Upham, of Boston, who delivered a very interesting and scholarly address upon the subject of "Pleasures and Pains;" upon the conclusion of which the members and invited guests sat down to dinner. Among the guests of the evening were Harved C. Ernst, M.D., of the Harvard Medical School; Drs. Thomas H. Chandler and Charles A. Brackett, of the Dental School; Rev. Albert H Plumb, D.D., of Roxbury, and Rev. George A. Crawford, D.D., of Boston; the latter gentleman having formerly held for nineteen years the position of chaplain in the United States navy, and who took occasion, in the post-prandial exercises, to relate many of his experiences and observations, both on shipboard and in China, in which latter place much of his time and life had been spent.

The Society was fortunate in having among its guests Dr. Ernst, the distinguished bacteriologist, who has recently returned from Berlin with a supply of lymph for the treatment of tuberculosis. The doctor expressed, with much clearness and force, his opinion as to the present and ultimate effects of the parataloid upon patients at present undergoing treatment in the Massachusetts General Hospital, and urged upon the dental profession, in view of the ease with which a localization of the disease may be effected in the pharynx, upon the tongue, and other portions of the oral cavity, by inoculation through carelessness on the part of the operator with the bacillus of tuberculosis, the importance of the most scrupulous care in the thorough cleansing of all instruments after an operation.

Subsequent to the after-dinner speaking, which at the annual meeting is always replete with wit and wisdom, the members throw aside for the time being all thoughts and cares of a busy every-day practice, and, after the fashion of the numerous societies in the academic department of the University upon the occasion of a dinner at this well-known hostelry, indulge in college songs until the lateness of the hour calls to mind the fact that all good times must have an ending.

Previous to adjournment, the following officers were elected for the ensuing year: President, Jere E. Stanton, M.D., D.M.D., of Boston; Recording Secretary, Waldo E. Boardman, D.M.D., of Boston; Corresponding Secretary, Charles H. Taft, D.M.D., of Cambridge; Treasurer, Dwight M. Clapp, D.M.D., of Boston.

*Executive Committee*: Waldo E. Boardman, D.M.D., of Boston; Herbert M. Clifford, D.M.D., of Boston; W. E. Page, D.M.D., of Boston.

*Editor.*—Henry L. Upham, D.M.D., of Boston.

Dr. H. W. Gillett, of Newport, R. I., was chosen to deliver the address at the next annual meeting.

Charles H. Taft,
*Corresponding Secretary.*

